# Efficient self-assembly and protective efficacy of infectious bursal disease virus-like particles by a recombinant baculovirus co-expressing precursor polyprotein and VP4

**DOI:** 10.1186/s12985-015-0403-4

**Published:** 2015-10-26

**Authors:** Hyun-Jeong Lee, Ji-Ye Kim, Soo-jeong Kye, Hee-Jung Seul, Suk-Chan Jung, Kang-Seuk Choi

**Affiliations:** Avian Disease Division, Animal and Plant Quarantine Agency, 175 Anyangro, Anyang, Gyeonggi 430-757 Republic of Korea

**Keywords:** Infectious bursal disease, Virus-like particle, Polyprotein, VP4, Vaccine

## Abstract

**Background:**

Virus-like particle (VLP) technology is considered one of the most promising approaches in animal vaccines, due to the intrinsic immunogenic properties as well as high safety profile of VLPs. In this study, we developed a VLP vaccine against infectious bursal disease virus (IBDV), which causes morbidity and mortality in chickens, by expressing a baculovirus in insect cells.

**Methods:**

To improve the self-proteolytic processing of precursor polyprotein (PP), we constructed a recombinant baculovirus transfer vector that co-expresses PP and the VP4 protease gene of IBDV.

**Results:**

Expression and VLP assembly of recombinant proteins and antigenicity of the VLP were examined by Western blotting, ELISA, and transmission electron microscopy. In animal experiments, vaccination with the recombinant VLP induced strong and uniform humoral immunity and provided complete protection against challenge with very virulent (vv) IBDV in SPF chickens (*n* = 12). As determined by the bursa of Fabricius (BF)/body weight (B/BW) ratio, the protection against post-challenge bursal atrophy was significantly higher (*P* < 0.001) in VLP-vaccinated birds than in non-vaccinated controls.

**Conclusions:**

Since the protective efficacy of the VLP vaccine was comparable to that of a commercially available inactivated vaccine, the recombinant VLP merits further investigation as an alternative means of protection against vvIBD.

## Background

Infectious bursal disease virus (IBDV) causes clinical signs, severe immunosuppression, and ultimately death in young chickens less than 6 weeks old. In particular, it destroys immune cells within the bursa of Fabricius (BF), resulting in decreased immune response to secondary infections and to vaccines for other diseases. IBDV belongs to the genus *Avibirnavirus* of the family *Birnaviridae*, whose members contain two double-stranded RNA genome segments (A and B) [1]. Genome segment B (approximately 2.9 kb) encodes viral protein 1 (VP1), the RNA-dependent RNA polymerase (RdRp) [2]. Genome segment A (approximately 3.3 kb) has two open reading frames (ORFs). The smaller ORF of the segment is approximately 400 bp long and encodes VP5 [3]. The larger ORF is approximately 3 kb in length and encodes precursor polyprotein (PP) with a molecular mass of 109 kDa. Nascent PP is cleaved into the individual viral proteins VP2, VP3, and VP4 [4]. VP2, in particular, is highly conformation-dependent with neutralizing epitopes [5–7], and so plays an important role in inducing protective immunity in the host [8–10].

In the global poultry industry, the control of IBDV is based mainly on the immunization of chickens with live, inactivated, or recombinant vaccines [11]. Traditionally, breeder flocks are immunized with an oil-emulsion inactivated IBDV vaccine after priming with live IBDV vaccines. This strategy gives protective passive immunity to the newly hatched chicks during the first few weeks of life. In many cases, oil-emulsion inactivated vaccines are manufactured using BF-derived IBDV antigen extracted from specific pathogen-free (SPF) chicks infected with virulent IBDV, since it is considered to be more immunogenic than egg-based or cell culture-based IBDV antigen. However, this process is time-consuming and requires the handling of infectious virus and a large number of SPF animals.

To overcome these limitations, various approaches for producing recombinant IBDV proteins *in vitro* have been developed to create alternative vaccines to the killed IBDV vaccine. Subunit vaccines carrying the protective VP2 of IBDV have been developed using recombinant proteins or vectored viruses including *E. coli* [12, 13], yeast [14–17], adenovirus [18], fowlpox virus [19], baculovirus [10, 20, 21], vaccinia virus [22], herpesvirus [23] and even plants [24–26], which avoid the safety issues associated with use of animals for vaccine production. However, the protection afforded by the VP2 vaccine tends to vary depending on the expression system employed and only a limited number of VP2 vaccines have been commercialized.

One form of novel vaccine carrying protective viral proteins is the virus-like particle (VLP). VLPs are assembled from viral structural proteins and resemble the structure of an authentic viral particle but are devoid of any genetic material. Therefore, recombinant vaccines based on VLP technology hold great promise for the development of highly efficacious vaccines that could replace inactivated vaccines directed against several pathogenic viruses of human and animals [27–30].

Regarding IBDV, one strategy for producing VLPs is the expression of PP, where the VP4 protease drives the maturation process of VP2 and VP3, which self-assemble into VLPs [20, 22, 31, 32]. However, the production of VLPs resembling authentic IBDV has proved to be unsatisfactory due to inefficient processing and maturation [31–34]. Another approach involves the co-expression of two structural proteins pVP2 and VP3 using two different recombinant baculoviruses [34–36], but a precise adjustment of the MOI for both viruses is required for efficient assembly of the VLPs [37].

Previously, we succeeded in producing swine vesicular disease virus VLPs by use of a single recombinant baculovirus bi-directionally expressing the viral protease (3CD) and structural precursor protein (P1) [38]. In this study, we applied similar strategy to produce IBDV VLPs by bi-directional co-expression of PP and viral protease VP4, which has not been attempted for IBDV. The expressed proteins were examined for morphology, antigenicity, immunogenicity, and protective efficacy.

## Materials and methods

### Virus

IBDV strain Kr/LC/2010 (LC10), kept in our laboratory, was used as a genetic template to obtain viral RNA or as a challenge virus for protection efficacy tests. LC10 is a very virulent IBDV (vvIBDV) isolated from a Korean broiler farm that had had an IBD outbreak in 2010. The virus was propagated in 9-day-old embryonated chicken eggs from SPF hens. The viral titer was expressed as 50 % embryo infective dose (EID_50_).

### Construction of a baculovirus transfer vector

**Fig. 1:**
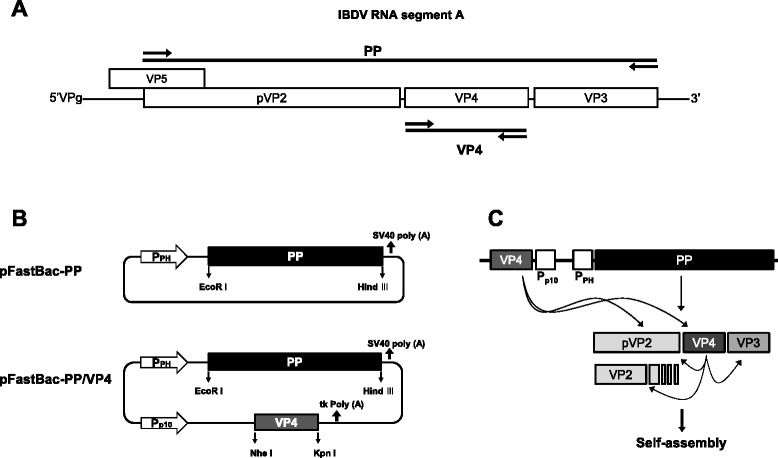
Strategy for construction of recombinant baculovirus transfer vector. **a** A schematic representation of the IBDV segment A showing regions amplified (solid lines) and primer positions (arrows). Amplification of PP and VP4 genes of IBDV was performed by RT-PCR. **b** Construction of a recombinant single (pFastBac-PP) or dual (pFastBac-PP/VP4) expression vector containing the PP and VP4 genes of IBDV. **c** A schematic of the strategy used for the production of VLP antigen induced by recombinant baculovirus Bac-PP/VP4 in Sf9 cells

The strategy for amplifying of the PP and VP4 genes of IBDV is outlined in Fig. 1a. Viral genomic RNA was extracted from LC10 using an RNeasy Mini kit (Qiagen, USA) and the PP and VP4 genes were separately amplified using an OneStep RT-PCR kit (Qiagen) according to the manufacturer’s instructions. Two PCR primer sets were designed to amplify the full-length ORFs of PP gene (3039 bp in length) and VP4 gene (813 bp in length), respectively (Table 1). Restriction enzyme sites were incorporated at the 5′ ends of the primers to facilitate cloning. The PCR products were purified and cloned separately into pGEM-T Easy (Promega). Then, the PP and VP4 inserts were excised from the vectors using restriction enzymes *EcoR* I and *Hind* III, and *Nhe* I and *Kpn* I, respectively, and both were sub-cloned into pFastBacDual vector (Invitrogen), where the PP gene was inserted downstream of the polyhedron promoter (P_PH_) and the VP4 gene was placed downstream of the P_p10_ promoter. For comparison purposes, the PP gene alone was inserted downstream of P_PH_ of pFastBac1 vector (Invitrogen). The resulting transfer plasmids containing both genes (PP and VP4) or the PP gene alone were designated pFastBac-PP/VP4 and pFastBac-PP, respectively (Fig. 1b).

**Table 1:** Primers used in this study

Genes	Primer name	Sequence^a^
Precursor polyprotein (PP)	LCPP-F	5′-CGG AAT TCC Gat gGAC AAA CCT GCA AGA TC-3′
	LCPP-R	5′-CCA AGC TTG GTC ACT CAA GGT CCT CAT-3′
VP4	LCVP4-F	5′-CGG CTA GCC Gat gAG GAT AGC TGT G-3′
	LCVP4	5′-GGG GTA CCC CTC ATT TGA TAA ACG TCG C-3′

^a^Lower case letters indicate ATG codons. Underlined sequences indicate the restriction enzyme sites

**Table 2:** Protective efficacy of recombinant VLP vaccine against challenge with vvIBDV in 3-week-old SPF chickens compared with a commercial inactivated vaccine or no vaccine (PBS treatment). Two weeks after immunization, birds were challenged with vvIBDV LC10 strain orally (10^4.5^EID_50_ per bird) and observed for 10 days

Vaccine	Mortality, n (%)	Clinical signs, n^a^	Gross lesions, n^b^	Bursal atrophy, n^c^	B/BW ratio, mean (SD)
Inactivated vaccine (*n* = 12)	0 (0)^**^	0^***^	3^**^	3^**^	3.46 (1.42)*
VLP vaccine (*n* = 12)	0 (0)^**^	0^***^	4^*^	3^**^	3.69 (1.74)**
Control (*n* = 12)	7 (58)	12	5	5	1.14 (0.30)

^a^Number of birds with clinical signs, including depression, anorexia, diarrhea, and death

^b^Number of surviving birds with gross lesions in the BF

^c^Number of surviving animals with a B/BW ratio (bursal weight/body weight x 1000) lower than 2

*P < 0.1, ** *P* <0.01, *** *P* <0.001 compared with the non-vaccinated control group (Fisher’s exact test)

### Rescue of recombinant baculoviruses

Recombinant baculovirus was generated using the Bac-to-Bac expression system (Invitrogen). Briefly, a recombinant DNA bacmid containing the PP and VP4 genes (bacmid-PP/VP4) was generated by transforming competent DH10Bac *E. coli* cells (Invitrogen) with the pFastBac-PP/VP4 according to the manufacturer’s instructions. The bacmid-PP/VP4 was then transfected into *Spodoptera frugiperda* 9 (Sf9) cells (2 × 10^6^ cells/ml) supplemented with Sf900 II medium (Gibco) in 6-well tissue culture plates. After 72 h incubation at 27 °C, the culture supernatants were subjected to a plaque assay to isolate recombinant baculovirus co-expressing PP and VP4 genes (rBac-PP/VP4). The presence of two inserts in the rBac-PP/VP4 was confirmed by PCR using the primer pairs described above. The positive plaque clone was designated rBac-PP/VP4. For comparison, a recombinant DNA bacmid containing the PP gene alone (bacmid-PP) and a recombinant baculovirus expressing PP alone (rBac-PP) were also generated using the pFastBac-PP in the same way as above. The recovered baculovirus preparations were stored at 4 °C.

### Preparation of recombinant proteins

Sf9 cells (5 × 10^5^ cells/ml) were seeded in a spinner flask (200 ml) containing Sf900II medium and were infected with rBac-PP/VP4 at a MOI of 1. Following a 3 day incubation, the infected cells were collected and precipitated by low-speed centrifugation (2,000 x g, 20 min). The cell pellets were re-suspended in 1/20 volume of ice-cold lysis buffer (10 mM Tris, 130 mM NaCl, 1 % (v/v) Triton X-100, pH 7.5) containing a protease inhibitor cocktail (BD Biosciences, USA), sonicated briefly with three 5 s pulses (Soniprep 150, MSE Sanyo, Japan), and clarified by high-speed centrifugation (11,650 x g, 35 min). The clarified supernatant was used as a recombinant antigen in this study. Recombinant proteins from rBac-PP were produced in the same manner.

### Western blotting

Expression of IBDV proteins in VLPs was confirmed by Western blotting. Briefly, proteins were separated on 10 % SDS-PAGE gels and blotted onto PVDF membranes (Life Technologies, USA). Membranes were incubated with anti-IBDV serum from chickens that had been immunized with purified, inactivated LC10. After several washes with 0.05 % Tween 20 in phosphate-buffered saline (PBS), bound antibodies were visualized by the use of alkaline phosphatase-conjugated goat anti-chicken IgG (H + L) (KPL, USA) and BCIP/NBT phosphatase substrate (KPL, USA).

### Double antibody sandwich enzyme-linked immunosorbent assay (DAS-ELISA)

For antigen quantification and to compare the antigenicity of the recombinant VP2 protein, we performed DAS-ELISA as described previously [39]. Briefly, MaxiSorp ELISA plates (Nunc) were coated with 50 μl of anti-VP2 monoclonal antibody (MAb) R63 [40] at the optimal concentration (4 μg/ml) in 0.01 M PBS at 37 °C for 1 h with constant shaking. The plates were washed three times with PBS containing 0.05 % Tween 20 (PBST) and then incubated with 50 μl of IBDV or VLP antigens for 1 h at 37 °C. Following a washing step, the plates were incubated for 1 h at 37 °C with 50 μl of anti-IBDV chicken sera (for VP2 quantification) or 10 chicken sera with various titers (for antigenic comparison) in blocking buffer (PBS containing 0.02 % Tween 20 and 1 % skimmed milk). To enable comparisons of antigenicity, SPF chicken sera and anti-IBDV chicken sera were also tested as a negative control and as a positive control, respectively. Following an additional washing step, the plates were incubated with peroxidase-conjugated goat anti-chicken IgG (H + L) for 1 h at 37 °C. After the final wash, the plates were incubated with 50 μl of tetramethylbenzidine (TMB) substrate solution (KPL, USA) for 10 min. The color development was stopped by 50 μl of 1 N HCl solution. The optical density (OD) was measured at 450 nm using an automatic ELISA reader (Tecan, Austria). For antigenic comparisons, data were normalized against those of negative sera and expressed as signal-to-positive (S/P) ratios using the formula (OD_sample_ – OD_negative_)/ (OD_positive_ – OD_negative_)x1000.

### Transmission electron microscopy

The rBac-PP/VP4-derived recombinant protein samples were purified by sucrose density gradient ultracentrifugation (89,454 x g, 6 h). The resulting fractions were fixed with 2.5 % glutaraldehyde in PBS (pH 7.2) and post-fixed in a solution of 1 % osmium tetroxide in PBS at 4 °C for 2 h. After dehydration in a graded series of ethanol and propylene oxide, the samples were embedded in spur epoxy resin. Ultrathin sections were made and stained with uranyl acetate and lead citrate, and were observed under an H-7100FA transmission electron microscope (Hitachi, Japan).

### Preparation of a pilot IBDV VLP vaccine

The rBac-PP/VP4-derived recombinant protein was used to make a pilot IBDV VLP vaccine. For this purpose, the recombinant protein was two-fold diluted and titrated using a commercial IBDV immunochromatographic (IC) kit (BioNote, Korea) with a detection limit for IBDV of between 10^3.1^ and 10^3.9^ EID_50_/ml [41]. The IC unit was the reciprocal of the highest dilution that gives a positive signal for IBDV. The pilot VLP vaccine was prepared by emulsifying 32 (2^5^) IC units of the antigen with Montanide ISA70 adjuvant (Seppic, France) at a ratio of 30:70 (v/v).

### Animal experimentation

A total of 36 SPF white leghorn chickens of 3 weeks of age (Namduck Sanitec, Korea) were used in the study. All animal procedures were approved and supervised by the Institutional Animal Care & Use Committee (IACUC) of the Animal and Plant Quarantine Agency (QIA). A first group of 12 birds was vaccinated with one dose of the pilot VLP vaccine (32 IC units in 0.5 ml per bird) via intramuscular route. A second group of 12 birds was injected intramuscularly with one dose (0.5 ml) of a commercial IBD vaccine (Nobilis IB + G + ND, MSD Animal Health). Finally, a last group of 12 control birds was injected with PBS. Blood samples were taken from all birds on day 14 after vaccination, and IBDV-specific serum antibodies were titrated with the use of IBD-XR ELISA kit (IDEXX, USA) according to the manufacturer’s instructions.

Two weeks after vaccination, all birds were challenged with 10^4.5^ EID_50_ (0.1 ml per bird) of vvIBDV LC10 strain via the oral route. The birds were monitored daily for overt clinical signs for 10 days after the challenge. Surviving birds were humanely sacrificed and examined the presence of gross BF lesions. The BF and body weights of each sacrificed bird were recorded. The BF/body weight (B/BW) ratio was calculated as the BF weight (g)/body weight (g) x 1,000. B/BW ratios < 2 were taken to indicate bursal atrophy.

### Statistical analyses

Statistical analysis was performed using GraphPad Instat version 3.05 for Windows (GraphPad Software). A two-tailed Fisher’s exact test was used to compare mortality, clinical signs, the presence of gross lesions, and B/BW ratios among groups. Differences in ELISA titer and mean B/BW ratio between groups were analyzed by one-way analysis of variance (ANOVA) with a Turkey-Kramer post hoc test. Linear regression was used to determine the strength of the correlation between the VLP antigen and IBDV antigen. *P* values less 0.05 were considered to indicate significance.

## Results

### Recombinant baculovirus containing the IBDV PP and VP4 genes

The PP and VP4 genes of IBDV were separately amplified and cloned into the pFastBacDual vector where they were placed under control of two different promoters. The recombinant baculovirus transfer vector was designated pFastBac-PP/VP4. For comparison purposes, the PP gene alone was cloned into the pFastBac1 vector. These constructs were confirmed to be in the correct orientation and to contain uninterrupted ORFs by PCR and sequencing (data not shown). pFastBac-PP/VP4 and pFastBac-PP were used to transform *E. coli* for the production of bacmids, which were then transfected into Sf9 cells. The transfected cells showed typical cytopathic effects, including low cell density, enlarged cells, and poor adherence to the substrate. Recombinant baculovirus particles were recovered from Sf9 cells and confirmed by PCR (data not shown).

### Analysis of expressed recombinant proteins

PP is known to be processed into VP2, VP3, and VP4 during viral replication in susceptible cells, and the resulting VP2 and VP3 self-assemble to form virion capsids (Fig. 1c). To investigate whether the rBac-PP/VP4-expressed PP also splits into VP2, VP3, and VP4, the recombinant proteins were biochemically analyzed. For this purpose, Sf9 cells were infected with either rBac-PP or rBac-PP/VP4 and recombinant proteins were extracted after 72 h.

**Fig. 2:**
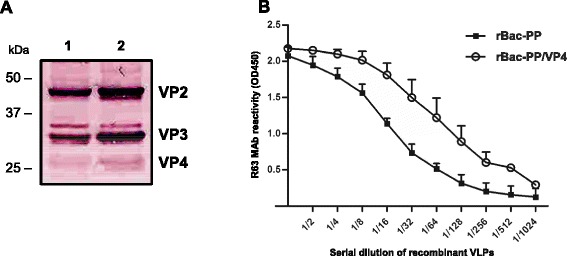
Biochemical analysis of proteins expressed by the recombinant baculovirus. **a** Western blot analysis of VLP antigens: lane 1, VLP antigen produced by rBac-PP; lane 2, VLP antigen produced by rBac-PP/VP4, **b** Antigenic reactivity of VLP antigens measured by DAS-ELISA. VLP antigens produced by rBac-PP and rBac-PP/VP4 were independently applied to DAS-ELISA to compare their R63 MAb reactivity with the R63 MAb reactivity of VP2 of IBDV. Serial dilution of VLP antigens were used to measure the titers of the expressed VP2 protein in VLPs

In Western blotting analysis, a faint protein band of 28 kDa, corresponding to VP4, was detected with anti-IBDV chicken serum in rBac-PP/VP4 protein extracts (Fig. 2a, right lane). Two bands of approximately 32 kDa and 42 kDa, corresponding to VP3 and VP2, respectively, were detected in both preparations, but the intensity of both bands (especially VP3) was lower for rBac-PP (Fig. 2a, left lane) than for rBac-PP/VP4. In DAS-ELISA, the yield of VP2 from rBac-PP/VP4 was at least four-times higher than that from rBac-PP (Fig. 2b). These findings indicate that the expression of recombinant proteins by rBac-PP/VP4 results in the cleavage of PP into VP2, VP3, and VP4, and that the cleavage occurs more efficiently when PP and VP4 are simultaneously expressed than when PP is expressed alone.

**Fig. 3:**
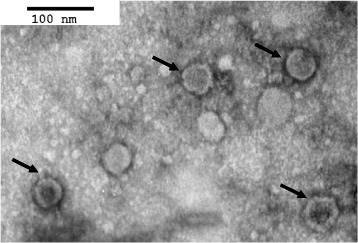
Electron microscopy of negatively stained recombinant VLPs purified from Bac-PP/VP4 infected Sf9 cells. VLPs are indicated by black arrows. Bar = 100 nm

To further investigate whether protein expression by rBac-PP/VP4 results in VLP formation, we used transmission electron microscopy (TEM). TEM examination of negatively stained protein preparations revealed the presence of numerous VLPs (Fig. 3). The VLPs had an authentic IBDV structure in terms of morphology, and the size of the particles was approximately 60 nm [42, 43]. No VLPs were observed in extracts from non-transfected Sf9 cells (data not shown).

**Fig. 4:**
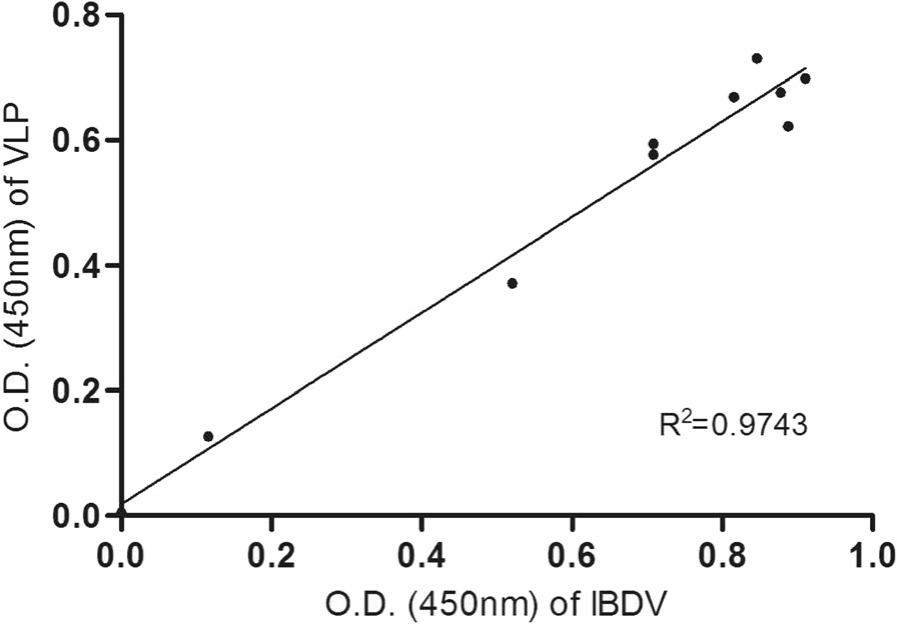
Correlation between recombinant VLP antigen reactivity and IBDV antigen reactivity. Linear regression of the mean S/P ratios obtained from the absorbance values of the reactivity of anti-VP2 MAb to wild type parental IBDV (LC10) and recombinant VLP antigen. The solid line represents the line of best fit

DAS-ELISA was also used to investigate whether VLPs are antigenically similar to IBDV. Ten chicken sera having different antibody titers against IBDV were tested for reactivity to both antigens at least three times. A strong linear association (*r*^*2*^ = 0.9594, *P* < 0.001) was observed for the reactivity between the parental LC10 strain and recombinant VLP antigen (Fig. 4).

### Immune response to the pilot IBDV VLP vaccine

**Fig. 5:**
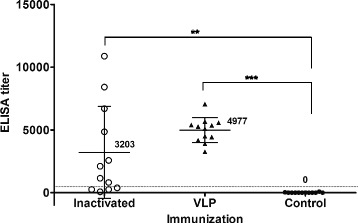
Humoral immune response to IBDV after VLP vaccination in chickens. Mean ELISA titers induced in SPF chickens after the injection of VLP vaccine or a commercial inactivated IBD vaccine. Error bars. standard deviations; dashed-line, cut-off titer (396). ^**^ *p* < 0.01 and ^***^ *p* < 0.001 by ANOVA with Tukey-Kramer post-test compared with the non-vaccinated control group

Then we investigated whether the VLP antigen provokes a protective immune response in chickens. For this purpose, a pilot IBDV VLP vaccine was formulated by emulsifying the VLP antigen at a final concentration of 32 IC units per dose in Montanide ISA70 adjuvant, pre-determined minimal effective dose of protective antigen in preliminary study. For comparison, a commercial inactivated oil-emulsion IBDV vaccine was used. Prior to immunization, all birds were found free of antibodies to IBDV when tested by ELISA (data not shown). Two weeks after a single immunization (Fig. 5), all 12 birds that had received the pilot VLP vaccine showed seroconversion to IBDV, with a mean ELISA titer of 4977 (SD = 993; range, 3259 to 7060). The commercial vaccine also induced an immune response to IBDV, but four of 12 vaccinated birds were still IBDV antibody-negative (ELISA titer, < 396). The mean ELISA titer in this group (3203) was lower than that achieved with the pilot VLP vaccine, and the variation (SD = 3661) was greater. Control birds that received only PBS remained IBDV antibody-negative during the experiment.

To test the protective efficacy of the vaccine, the vaccinated birds were challenged with vvIBDV strain LC10 (10^4.5^ EID_50_ per bird) after 2 weeks, and clinical signs and mortality were observed for 10 days. As shown in Table 2, all 12 control birds (treated just with PBS) showed severe clinical signs such as depression, anorexia, and diarrhea, and 7 (58 %) died within 10 days. Pathological examination revealed BF lesions, such as caseous cores and petechial hemorrhage, and bursal atrophy. In contrast, all 12 birds that had been vaccinated with the pilot VLP vaccine showed no clinical signs and survived the vvIBDV challenge. Pathological examination found no gross lesions in the BF of eight of these birds (67 %), while four did have bursal lesions, including bursal atrophy in three animals. Similar results were obtained in the 12 birds vaccinated with the commercial IBDV vaccine: they showed no clinical signs, and three birds had pathological bursal lesions and bursal atrophy. Among them, one had no detectable antibodies, while the other two had low level of antibodies (ELISA titers < 1200).

## Discussion

In this study, we employed a novel strategy to produce VLPs based on a single recombinant baculovirus that simultaneously expresses PP and VP4 under the control of different promoters. Western blot analysis and DAS-ELISA revealed that PP expressed by rBac-PP/VP4 and rBac-PP was successfully split into structural proteins VP2, VP3, and VP4. Notably, the yields of VP2 and VP3 were greater when VP4 was co-expressed with PP. Since VP2 and VP3 are believed to form, respectively, the outer and inner layers of the virion in roughly equimolar amounts [44, 45], increases in the concentration of both proteins likely assist in forming VLPs. This was supported by the TEM observation that rBac-PP/VP4-expressed proteins formed VLPs with a diameter of 60 nm, resembling authentic virus. Until now, however, PP expression alone has often resulted in inefficient assembly of VLP and most expressed proteins formed tubule-like structures instead of VLPs [20]. Moreover, insect cells lack the puromycin-sensitive aminopeptidase (PurSA) that, in host cells, is involved with virus assembly [46]. Thus, PP expression alone is inadequate to produce VLPs efficiently in complete form. Nevertheless here we successfully produced VLPs from PP expressed by recombinant baculovirus in insect cells through co-expression of VP4 protease. Similarly, the use of a highly productive baculovirus transfer vector pAcYM1 also seems to be involved in enhanced assembly efficiency of the VLPs [34]. Therefore, an increased yield of VP4 possibly improves both the yield of VP2 and VP3 and the efficiency of VLP formation. With the observation that VP3 is crucial to the formation of a VLP [32], it can be also suggested that the high VP3 expression in this study had a positive impact on VLP formation.

It is known that the major neutralizing sites on VP2 are conformation-dependent, and that incorrect VP2 folding results in a lack of immunogenicity in chickens [9, 20, 47]. This means that the immunogenicity and protective capability of the VLP can be greatly affected by antigenic structure of VP2 in VLPs. Recently, Jackwood *et al*. [35] demonstrated that recombinant VLPs formed by co-expression of VP2 and VP3 are more close in antigenic structure to naïve IBDV VP2 than to pVP2 alone when analyzed using VP2-specific monoclonal antibodies. The VLPs produced here also showed antigenicity close to naïve IBDV when tested by DAS-ELISA and a commercial IC kit. We postulate that co-expression of VP4 protease with PP increased the yield of VP2 and VP3 by strengthening the proteolytic processing of IBDV PP, followed by proper morphogenesis of the VLPs resulting from post-translational modifications.

The VLP vaccine elicited a strong humoral immune response and protected the chickens completely from vvIBDV infection in SPF chickens, in contrast to previous results where recombinant IBDV PP alone induced incomplete protection [20]. The difference might be due to the correct assembly and antigenic structure of the VLPs resembling authentic IBDV in our study. In part, baculovirus itself contained in the VLP vaccine of the study is likely to enhance immunogenicity by activating local innate immune response [48]. Besides, the normal poultry vaccine program primes young birds with live attenuated IBDV vaccines and then hyperimmunizes them with killed IBDV vaccine several weeks before they lay in most breeder farms. Therefore, it is necessary to determine whether the combination of VLP vaccine with live attenuated vaccine on the farm could provoke a more solid protective immunity in breeder flocks than the VLP vaccine alone. If so, our VLP vaccine could be an alternative to the commercial killed IBDV vaccine (BF-derived IBDV antigen).

Moreover, genomic RNAs from vvIBDV were used as the genetic source for the production of the VLPs, thus the antigenic structure of our VLPs more likely resembles vvIBDV than that of the current vaccine strain. If this is true, VLP vaccination in breeder flocks could provide progeny chicks with efficacious protective immunity via maternal-derived antibodies (MDAs), which are critical for early protection. The success of live attenuated IBDV vaccination in young chicks with MDAs is greatly affected by the degree of flock MDA uniformity as well as MDA titers. MDA uniformity is the result of the breeder’s immunity induced by IBDV vaccination, exposure to field virus, immune suppression, and age of the flock. In particular, the MDA uniformity of a flock is critical to determine the optimal timing and frequency of live attenuated IBDV vaccination in young chicks. Practically, a flock with poor MDA uniformity (e.g., a coefficient of variation >80 %) requires a sophisticated and complicated live attenuated IBDV vaccination program with several vaccinations in order to protect birds from exposure to vvIBDV [13, 49]. Thus, it is noteworthy that the VLP vaccine developed here induced a more uniform antibody response in a flock than did the commercial killed IBDV vaccine. If this finding is confirmed, breeder flocks vaccinated with the VLP vaccine will be able to confer uniform MDA titers to progeny chicks, which would be an advantage in deciding the optimal timing for IBD vaccination in young offspring flocks.

## Conclusions

To improve the self-proteolytic processing of PP of IBDV, VP4 protease of IBDV were co-expressed with the PP in insect cells by single recombinant baculovirus containing the PP and VP4 protein genes. Simultaneous expression of PP and VP4 protease recombinant proteins resulted in increase in the yields of VP2 and VP3, thus possibly leading to the efficient formation of VLPs morphologically and antigenically similar to IBDV. In animal experiment, protective efficacy of our VLP vaccine was comparable to that of the commercial killed IBDV vaccine. In conclusion, IBD VLP vaccine in this study protects chickens from vvIBDV that is possibly comparable to commercial vaccines and possibly due to the presence of the additional VP4.
